# *Cupriavidus pinatubonensis* JMP134 Alleviates Sulfane Sulfur Toxicity after the Loss of Sulfane Dehydrogenase through Oxidation by Persulfide Dioxygenase and Hydrogen Sulfide Release

**DOI:** 10.3390/metabo13020218

**Published:** 2023-02-02

**Authors:** Yufeng Xin, Yaxin Wang, Honglin Zhang, Yu Wu, Yongzhen Xia, Huanjie Li, Xiaohua Qu

**Affiliations:** 1 College of Life Sciences, Qufu Normal University, Qufu 273165, China; 2 State Key Laboratory of Microbial Technology, Shandong University, Qingdao 266237, China; 3 School of Medicine, Cheeloo College of Medicine, Shandong University, Jinan 250012, China

**Keywords:** thiosulfate, sulfane sulfur, oxidation, the Sox system, persulfide dioxygenase, glutathione, bacteria

## Abstract

An incomplete Sox system lacking sulfane dehydrogenase SoxCD may produce and accumulate sulfane sulfur when oxidizing thiosulfate. However, how bacteria alleviate the pressure of sulfane sulfur accumulation remains largely unclear. In this study, we focused on the bacterium *Cupriavidus pinatubonensis* JMP134, which contains a complete Sox system. When soxCD was deleted, this bacterium temporarily produced sulfane sulfur when oxidizing thiosulfate. Persulfide dioxygenase (PDO) in concert with glutathione oxidizes sulfane sulfur to sulfite. Sulfite can spontaneously react with extra persulfide glutathione (GSSH) to produce thiosulfate, which can feed into the incomplete Sox system again and be oxidized to sulfate. Furthermore, the deletion strain lacking PDO and SoxCD produced volatile H_2_S gas when oxidizing thiosulfate. By comparing the oxidized glutathione (GSSG) between the wild-type and deletion strains, we speculated that H_2_S is generated during the interaction between sulfane sulfur and the glutathione/oxidized glutathione (GSH/GSSG) redox couple, which may reduce the oxidative stress caused by the accumulation of sulfane sulfur in bacteria. Thus, PDO and H_2_S release play a critical role in alleviating sulfane sulfur toxicity after the loss of soxCD in *C. pinatubonensis* JMP134.

## 1. Introduction

**Figure 1:**
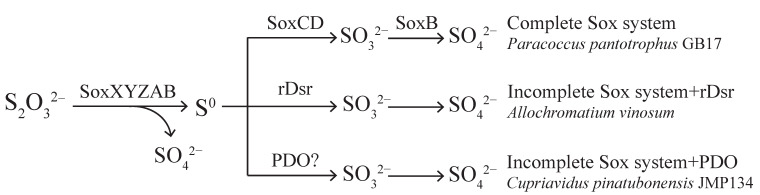
Currently known or proposed thiosulfate oxidation pathways related to the Sox system. There are two well known thiosulfate oxidation pathways related to the Sox system. The complete Sox system directly oxidizes thiosulfate to sulfate without intermediate production, where SoxCD is a sulfane dehydrogenase responsible for the oxidation of sulfane sulfur generated by the incomplete Sox system (SoxXA, SoxB, SoxYZ). Then, the oxidation product sulfite is oxidized to sulfate by SoxB. The Sox system widely exists in bacteria (top route). In another mechanism, rDsr (reversed dissimilatory (bi)sulfite reductase) oxidizes the sulfane sulfur produced by the incomplete Sox system to sulfite, which often exists in the phyla Proteobacteria and Chlorobi (middle route). Alternatively, this study proposed that PDO (persulfide dioxygenase) may work in concert with the incomplete Sox system to oxidize thiosulfate to sulfate, and PDO is widespread in bacteria (bottom route).

Thiosulfate is an important intermediate in sulfur oxidation and reduction [1,2,3], and it is usually oxidized to sulfate by microorganisms [4,5], which are some of the most important links in the biogeochemical sulfur cycle. The Sox multienzyme system is an important pathway for completely oxidizing thiosulfate to sulfate; however, sulfane sulfur production may occur during thiosulfate oxidation given the variability in each component of the Sox system among different species [6]. Sulfane sulfur at high levels was toxic to bacteria. For example, this was the case for *Escherichia coli* under both anaerobic and aerobic conditions [7]. Under oxic conditions, high sulfide concentrations can cause *Beggiatoa* sp. 35Flor death indirectly by inducing excessive sulfur accumulation [8]. Further, elemental sulfur has been used as an antimicrobial agent for ages, and its efficiency is likely impaired by its low solubility [9]. At present, the understanding of sulfane sulfur oxidation is limited to the reverse dissimilatory (bi)sulfite reductase (rDsr) system found in photoautotrophic sulfur oxidizers [10,11], the sulfane dehydrogenase (SoxCD) in the Sox system [12], and persulfide dioxygenase (PDO) (Figure 1).

The Sox system was studied in detail in the chemoautotrophic bacterium *Paracoccus pantotrophus* GB17 [13]. *Cupriavidus pinatubonensis* JMP134 contains a complete Sox system on chromosome A [3]. The complete Sox system consists of four enzymes, SoxYZ, SoxXA, SoxB, and SoxCD which are located in the periplasmic space of bacteria, encoded by seven genes, soxABCDXYZ [12]. The mechanism of thiosulfate oxidation by the Sox system is relatively clear. Assisted by the hemoprotein SoxXA [14], one molecule of the thiosulfate ion binds to a cysteine residue at the extreme C-terminus of the SoxY protein to generate SoxYZ-SSSO_3_^2−^ [15]. This intermediate is subsequently hydrolyzed by SoxB, releasing one molecule of sulfate and generating the intermediate SoxYZ-SS^−^ [16]. Then, SoxCD catalyzes the oxidation of SoxYZ-SS^−^ to sulfone (SoxYZ-SSO_3_^2−^) in a six-electron transfer reaction [17]. Next, SoxB hydrolyzes SoxYZ-SSO_3_^2−^, produces another molecule of sulfate, and releases SoxYZ-S^−^ to participate in the next round of thiosulfate oxidation. The Sox system finally yields 10 mol of H^+^ ions per mol thiosulfate, which is the only metabolic energy and reducing power for chemoautotrophic microorganisms [12].

Interestingly, many microorganisms possess incomplete Sox systems in nature, such as *Allochromatium vinosum*, *Thermotrix*, *Beggiatoa*, and *Thioalkalivibrio*. SoxCD is missing from the Sox system of these microorganisms [6]. When thiosulfate is oxidized by an incomplete Sox system, only two electrons and two H ions are released, and the sulfonic acid group of thiosulfate is converted to sulfate [13]. In addition, the sulfane sulfur of thiosulfate is converted to SoxYZ-SS^−^, which fails to be further oxidized. After a new round of thiosulfate oxidation, the sulfane chain on SoxYZ-SS^−^ is continuously extended. Finally, sulfur globules are generated and separated from SoxYZ-S^−^. The resulting sulfur globules are often located intra- or extracellularly in bacteria and are difficult to oxidize by most organisms due to their strong hydrophobicity [6]. The oxidation of sulfane sulfur is an important step in the process of thiosulfate oxidation by these microorganisms, and this process remains unclear in many microorganisms.

rDsr has been shown to oxidize sulfane generated from the oxidation of thiosulfate by the incomplete Sox system to sulfite in the purple sulfur bacterium *Allochromatium vinosum* [10]. The rDsr system usually consists of 15 open reading frames designated dsrABEFHCMKLJOPNRS. The sulfite reductase encoded by *dsrAB* is often used as a signature marker to evaluate the existence of the rDsr system [11]. The rDsr was detected in the phyla Proteobacteria (classes Alpha-, Beta-, Gamma- and Deltaproteobacteria) and Chlorobi [11]. However, rDsr does not exist in many bacteria that can oxidize thiosulfate, such as *C. pinatubonensis* JMP134 [3]. Unlike rDsr, PDOs are widespread in heterotrophic or chemoautotrophic bacteria and in the mitochondria of humans or plants [18,19,20]. In *C. pinatubonensis* JMP134, PDO is usually located in the cytoplasm [21]. PDO often works with sulfide:quinone oxidoreductase (SQR), which is located on the cytoplasmic side of the membrane in *C. pinatubonensis* JMP134 [21], to oxidize H_2_S to thiosulfate. In this process, SQR oxidizes H_2_S and produces polysulfides (S^0^) bound to the cysteine residue of SQR [22]. Glutathione (GSH), a sulfane receptor, spontaneously reacts with bound S^0^ to produce persulfide glutathione (GSSH), which is the real substrate of PDO [23]. GSSH is then oxidized by PDO to produce sulfite and consume oxygen [18]. Therefore, these bacteria which contained SQR and PDO, PDO together with GSH oxidize S^0^ bound by SQR to sulfite, which indicated an important H_2_S detoxification mechanism. Based on the above analysis, we assumed that PDO could alleviates the toxicity of sulfane sulfur accumulation resulting from the oxidation of thiosulfate by the incomplete Sox system.

To test our hypothesis, we selected the bacterium *C. pinatubonensis* JMP134 with a complete Sox system and PDO. Through traditional gene knockout, product analysis, gene transcription, and bioinformatics analysis, we revealed two pathways that alleviate the toxicity of sulfane sulfur in *C. pinatubonensis* JMP134. One is that sulfane sulfur is oxidized by PDO, and the other is that sulfane sulfur is released by conversion to hydrogen sulfide.

## 2. Materials and Methods

### 2.1. Bacterial Strains, Primers and Media

The method used to construct the deletion strains and complementation strains was described previously [3]. Briefly, the upstream and downstream fragments of the target gene obtained by PCR were ligated with the linearized plasmid pK18mobsacB by a modified in-fusion method [24] to construct a deletion plasmid, transforming the deletion plasmid into *E. coli* S17-1 and then transferring to *C. pinatubonensis* JMP134 by conjugation. The complementation strain was generated by transforming a recombinant plasmid into the corresponding mutant. The recombinant plasmid was constructed by assembling the PCR-amplified gene into the broad host plasmid pBBR1MCS2 (linearized via PCR) using a modified in-fusion method [24]. The correct deletion or complementation strain was confirmed by PCR. Details and all required primers used for deletion and complementation of strains are shown in Table 1 and Table 2, respectively. Lysogeny-Broth (LB) medium and mineral medium were used, as described in our previous report [3]. The carbon source was 0.5% (wt./vol.) monosodium glutamate when the mineral medium was used. All chemicals were purchased from Macklin Biochemical Co., Ltd. (Shanghai, China).

### 2.2. Thiosulfate Oxidation by Whole-Cell and Product Detection

*C. pinatubonensis* JMP134 and knockout strains were aerobically cultivated in LB medium with shaking at 200 rpm at 30 °C. When the optical density at 600 nm (*OD*_600_) reached 3.0, 1 mL of culture was transferred to 100 mL of fresh mineral medium, and the cells were further cultivated to *OD*_600_ = 1.5 at 30 °C. Then, 200 µM Na_2_S_2_O_3_ was added to the culture for induction of the Sox system and further cultivated to *OD*_600_ = 2.5~3.0. The cells were harvested by centrifugation (6000× *g*, 10 min) and resuspended in 100 mM HEPES buffer, pH 7.0, to a turbidity of 2 at 600 nm. One milliliter of the cell suspension was transferred to a 15-mL glass tube. Freshly prepared thiosulfate (1 mM) was added to initiate the reaction. The tube was closed with a butyl rubber stopper and incubated at 30 °C with gentle shaking. A lead acetate test strip was placed inside of the tube to monitor H_2_S gas. For the quantification of H_2_S, a zinc acetate absorbing method was developed. Briefly, in the above process of thiosulfate oxidation by whole cells, 500 μM zinc acetate and 1 mM thiosulfate were added together to initiate the reaction, and the other steps remained unchanged. At various time intervals, 1 mL of the reaction solution was centrifuged (12,000× *g*, 5 min), and the supernatant was removed. The precipitate was resuspended in 1 mL of deionized water, and sulfide was measured by using the methylene blue method [25]. The thiosulfate, sulfate, and cellular sulfane sulfur were analyzed at various time intervals. The detection of thiosulfate and sulfate was performed, as previously reported [3]. Briefly, the suspension was centrifuged (13,000× *g*, 3 min), and the supernatant was obtained for detection by using an ion chromatograph system (ICS) (Dionex ICS-1100, Sunnyvale, CA, USA). The ICS conditions were determined using a reported protocol [3]. Cellular sulfane sulfur, including polysulfides, persulfides (RS_2_H and RS^2−^), polypersulfides (RSnH and RSn^−^, *n* ≥ 3), organic polysulfides, and elemental sulfur [26,27], was detected by the cyanolysis method [26], with minor modification. Briefly, 250 μL of the cell suspension were transferred to a mixture of 550 μL of 1% boric acid and 200 μL of 100 mM cyanide, heated in boiling water for 5 min, and then cooled to room temperature. Next, 100 μL of ferric nitrate reagent were added. The sample was centrifuged to remove cell debris, and the *OD*_460_ was measured. When measuring the pH of the cell culture, the HEPES buffer was replaced with 1% physiological saline, and the other protocols remained unchanged. The pH was measured using a LE438 electrode (Mettler Toledo, Columbus, OH, USA) in a cell suspension. For GSSG detection in cell lysates, the cells from 20 mL of culture were harvested by centrifugation (6000× *g*, 5 min) at various time intervals and resuspended in 500 μL 100 mM pH 7.0 HEPES. The suspension was disrupted by boiling for 10 min. The lysate was centrifuged at 6000× *g* for 5 min to remove cell debris. GSSG in the cell lysate was determined using a GSSG Assay Kit (S0053, Beyotime, Shanghai, China), according to the manufacturer’s protocols. The chemical basis of the kit is to reduce GSSG to GSH by glutathione reductase in the presence of NADPH [28]. The microplate reader assay method for GSH by the sulfhydryl reagent 5,5′-dithio-bis(2-nitrobenzoic acid) (DTNB) to form the yellow derivative 5′-thio-2-nitrobenzoic acid (TNB), which is measurable at 412 nm. All analyses were performed in triplicate, and the results are expressed as the mean ± SD.

### 2.3. Real-Time Quantitative Reverse Transcription PCR (RT–qPCR)

For RT–qPCR, strains were grown in mineral medium with shaking at 200 rpm at 30 °C until the *OD*_600_ reached 0.5, and then 1 mM sodium thiosulfate was added to the medium. After 30 min of cultivation, the cells were collected by centrifugation (6000× *g*, 10 min), and RNA was extracted. Additionally, a culture without sodium thiosulfate served as the control. RNA samples were prepared using a Bacterial RNA Extraction Kit (R403-01, Vazyme, Nanjing, China). Total cDNA was synthesized using the HiScript^®^ III 1st Strand cDNA Synthesis Kit (R312-01/02, Vazyme). RT–qPCR was performed using ChamQ SYBR qPCR Master Mix (Q311-02, Vazyme) and a LightCycler 480II (Roche) system with an initial incubation at 95 °C for 120 s followed by 45 cycles of 10 s at 95 °C, 10 s at 52 °C, and 15 s at 72 °C. To calculate the relative expression levels of the tested genes, gyrA (glyceraldehyde-3-phosphate dehydrogenase) gene expression was used as the internal standard, and the quantification method (2^−ΔΔCt^) was the same as previously reported [29]. Each sample was repeated with three independent extractions of RNA, and the standard deviations (SDs) were calculated and are shown as error bars. All primers designed are listed in Table 2.

### 2.4. Bioinformatics Analysis

An assembly summary list of 19092 completed bacterial genomes was downloaded from the National Center for Biotechnology Information (NCBI) (https://ftp.ncbi.nlm.nih.gov/genomes/genbank (accessed on 26 June 2020)). One genome, the type strain when available from each species, was selected to reduce the number of genomes to 5013 (Table S1). The amino acid sequence for all genes of these 5013 species were downloaded to generate a local Basic Local Alignment Search Tool (BLAST) database using the makeblastdb script of BLAST+. The query amino acid sequences of SoxA, SoxB, SoxC, SoxD, SoxX, SoxY, and SoxZ were collected from the Kyoto Encyclopedia of Genes and Genomes (KEGG) database (https://www.genome.jp/kegg/kegg2.html (accessed on 26 June 2020)). The query amino acid sequences of PDO and DsrAB used for BLAST were collected from previous papers [1,11,30]. These amino acid sequences were used as query sequences for local BLASTp, which was performed by Blast+ (2.12.0) [31] with uniform criteria (e-value < 1 × 10^−3^, coverage ≥40%, percentage of identity ≥30%) in local BLAST database. The candidate homologous proteins collected via BLASTp were further filtered using conserved domain analysis performed by the Batch Web CD-Search Tool of NCBI (https://www.ncbi.nlm.nih.gov/Structure/bwrpsb/bwrpsb.cgi (accessed on 30 June 2020)). We noticed that SoxA contains the complete thiosulf_SoxA domain (TIGR04484). SoxB contains the complete thiosulf_SoxB domain (TIGR04486). SoxC contains the complete sulfite_DH_soxC domain (TIGR04555). SoxX contains the complete thiosulf_SoxX domain (TIGR04485). SoxY contains the complete thiosulf_SoxY domain (TIGR04488). SoxZ contains the complete SoxZ_true domain (TIGR04490). All known PDOs contain completed GloB (COG0491), Lactamase_B (smart00849), and POD-like_MBL-fold (cd07724). Therefore, these motifs were used as standard features for further filtration. Finally, the identified target proteins matched their taxonomic information according to their GenBank accession numbers by the Taxonkit toolkit [32]. The 16S rRNA gene sequences from 362 species containing the complete or incomplete Sox system were retrieved from the GenBank database. These sequences were aligned using Muscle [33]. A phylogenetic tree based on the 16S rRNA gene sequences was constructed using the neighbor-joining method with pairwise deletion, p-distance distribution, and bootstrap analysis of 1000 repeats as parameters in MEGA 11 [34]. The phylogenetic tree was visualized via the iTOL website (https://itol.embl.de (accessed on 6 July 2022)) [35].

### 2.5. Statistical Analysis

GraphPad Prism software version 9.0.0 (San Diego, CA, USA) was used for statistical analyses. The unpaired Student’s *t* test was used to assess the statistical significance when monitoring the pH and GSSG and to analyze the expression differences of genes. The data are reported as the means ± SDs. A value of *p* < 0.05 indicated statistical significance, and a value of *p* < 0.01 was considered highly significant.

## 3. Results

### 3.1. Thiosulfate Oxidation by Wild-Type and Mutant Strains of C. pinatubonensis JMP134

**Figure 2:**
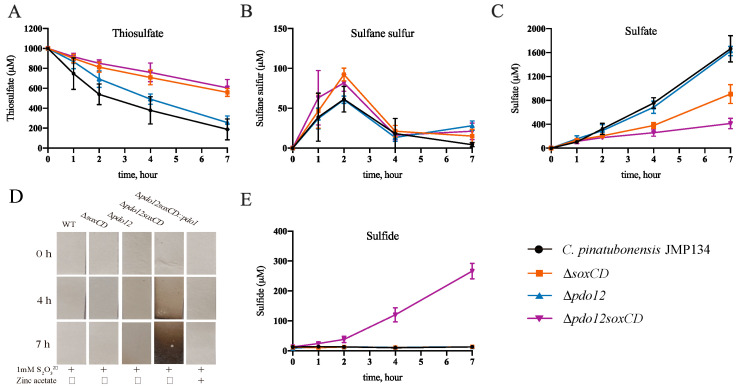
Thiosulfate oxidation by wild-type JMP134 and its mutants. Cells of *C. pinatubonensis* JMP134, Δ*soxCD*, Δ*pdo12soxCD*, and Δ*pdo12* were harvested, washed, and resuspended at an *OD*_600_ of 2.0 in 100 mM HEPES buffer, pH 7.0. Then, 1 mM thiosulfate was added to initiate the reaction. Thiosulfate (**A**), sulfane sulfur (**B**), sulfate (**C**), H_2_S gas (**D**), and dissolved sulfide (**E**) were determined at different time points. Except for the H_2_S gas values, all data are the average of at least three samples with SD (error bar).

The wild-type strain of *C. pinatubonensis* JMP134 oxidized approximately 800 μM thiosulfate within 7 h. Sulfane sulfur was gradually produced, reaching the highest value at 2 h (approximately 50 μM), after which the sulfane sulfur gradually decreased to almost undetectable levels at 7 h. Sulfate was continuously produced from the addition of thiosulfate. Finally, approximately 1600 μM sulfate was produced at 7 h with a molar ratio of consumed thiosulfate close to 2:1, indicating that all the consumed thiosulfate was oxidized to sulfate after 7 h. Two PDOs are expressed in *C. pinatubonensis* JMP134 [3], and the rate and product of thiosulfate oxidation of strain Δ*pdo12* were similar to those in the wild-type strain (Figure 2), indicating that PDO is not necessary for thiosulfate oxidation by the complete Sox system.

The rate of thiosulfate oxidation by the Δ*soxCD* strain was lower than that of the wild-type strain, and only approximately 400 μM thiosulfate was oxidized within 7 h. More sulfane sulfur (80 μM) accumulated at 2 h in the Δ*soxCD* strain compared with the wild-type strain. However, sulfane sulfur did not continue to accumulate as expected and slowly disappeared to almost undetectable levels at 7 h. The strain finally produced approximately 800 μM sulfate in 7 h, which is in mass balance with the consumed thiosulfate, indicating that all the consumed thiosulfate was also oxidized to sulfate. The reduced rates of thiosulfate oxidation in the mutant strains were partially recovered when the deleted genes were complemented in trans on a plasmid (Figure S1). This suggested that there are other enzymes which oxidize the sulfane sulfur accumulated due to the lack of SoxCD.

Furthermore, the products of thiosulfate oxidation by the quadruple knockout strain Δ*pdo12soxCD* were examined. This strain produced 80 μM sulfane sulfur at 2 h, and production was gradually reduced to zero (7 h) (Figure 2B). This finding is similar to that noted in the previous three strains. Surprisingly, this strain produced only approximately 400 μM sulfate in 7 h and consumed approximately 400 μM thiosulfate. If calculated exclusively based on sulfur, approximately 50% of sulfur was not oxidized to sulfate. To determine the whereabouts of this sulfur, we tested whether these strains volatilized H_2_S gas when oxidizing thiosulfate. The results of the lead acetate test clearly showed that the quadruple knockout strain produced a large amount of H_2_S, but no H_2_S was produced in the above three strains (Figure 2D). However, the complemental strain containing the *pdo* gene (Δ*pdo12soxCD::pdo1*) significantly reduced the volatility of H_2_S when oxidizing thiosulfate (Figure 2D). Together, these results indicated that PDO in this bacterium partially alleviated the loss of SoxCD in terms of conversion of thiosulfate to sulfate. Furthermore, when PDO and SoxCD are absent, H_2_S generation and volatilization seem to be the main way of sulfane sulfur release.

To quantify H_2_S gas production by the quadruple knockout strain, a method based on zinc acetate absorption was developed. The results showed that, after adding 500 μM zinc acetate to the HEPES (N-2-hydroxyethylpiperazine-N′-2-ethanesulfonic acid) buffer, the oxidation rate of thiosulfate, the production and consumption rate of sulfane sulfur, and the accumulation rate of sulfate were not affected when all strains oxidized thiosulfate. These findings are similar to the data presented in Figure 2A–C. However, the lead acetate test strip of the quadruple strain did not turn black (Figure 2D), indicating that H_2_S gas was absorbed as sulfide by zinc acetate. In addition, the production of sulfide of the quadruple strain was approximately 260 μM at 7 h, as detected by the methylene blue method, whereas no sulfide was detected in other strains (Figure 2E). Thus, the quadruple strain Δ*pdo12soxCD* oxidizes thiosulfate to sulfate and sulfide when zinc acetate is available in the buffer. These results suggested that the generation of H_2_S gas occurs in the absence of zinc acetate and accounts for the apparent missing sulfur in those experiments (Figure 2A–C).

Two pdo and soxC knockout strains, namely, Δ*pdo1soxCD* and Δ*pdo2soxCD*, were analyzed to determine which PDO oxidize the sulfane sulfur in thiosulfate oxidation. The results showed that neither of the two strains produced H_2_S gas (Figure S2), which indicated no sulfane sulfur accumulation in the two strains. Therefore, we hypothesized that the two PDOs are complementary in oxidizing sulfane sulfur produced by an incomplete Sox system.

### 3.2. Changes in pH Values When Bacteria Oxidize Thiosulfate

**Figure 3:**
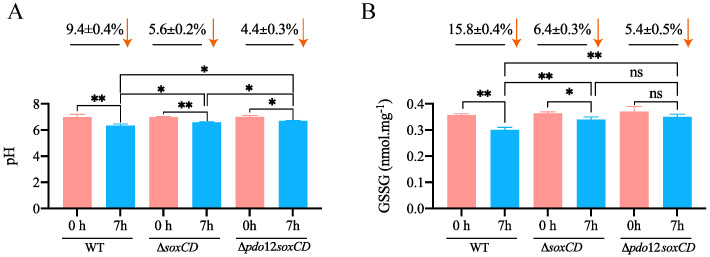
pH and GSSG detection of wild-type JMP134 and the mutants. Cells were harvested, washed, and resuspended at an *OD*_600_ of 2.0 in 100 mM HEPES buffer, pH 7.0. When measuring the pH, HEPES buffer was replaced with 1% physiological saline. Then, 1 mM thiosulfate was added to initiate the reaction. The pH of the suspensions was measured (**A**), and the GSSG of the cell lysate (**B**) was determined at 0 and 7 h. WT, wild-type *C. pinatubonensis* JMP134. *: *p* < 0.05; **: *p* < 0.01. ns: not significant.

In unbuffered medium, the pH value of wild-type *C. pinatubonensis* JMP134 and knockout strains decreased to varying degrees after adding thiosulfate (Figure 3A). The pH value of the wild-type strain decreased by approximately 9.4% at 7 h. Furthermore, the pH values of the Δ*soxCD* and quadruple knockout strains Δ*pdo12soxCD* decreased by 5.6% and 4.4%, respectively (Figure 3A), after 7 h. The pH of the Δ*pdo12soxCD* strain in which both *pdo* and *soxC* were knocked out was slightly higher than that of the Δ*soxCD* strain with knockout of *soxC* alone (Figure 3A). This finding is consistent with the observation that H_2_S gas volatilization occurs when thiosulfate is oxidized by Δ*pdo12soxCD* (Figure 2D), possibly because the volatilization of H_2_S acid gas will lead to the alkalization of the original environment [36].

### 3.3. GSSG Detection in the Cell Lysates

The function of PDO depends on GSH in vivo, and GSH/GSSG is an important redox couple in cells [37]. We detected the intracellular GSSG concentration of these strains when they oxidized thiosulfate. The GSSG in the wild-type strain gradually decreased by 15.8% after 7 h (Figure 3B). However, lower reduction of GSSG was noted in Δ*soxCD* (6.4%) compared with the wild-type strain (Figure 3B). Furthermore, the reduction in GSSG of Δ*pdo12soxCD* (5.4%) was less than that of Δ*soxCD* (Figure 3B), indicating that the strain that lacked PDO may have another source of GSSG.

### 3.4. Gene Expression in the Wild-Type and Mutant Strains

**Figure 4:**
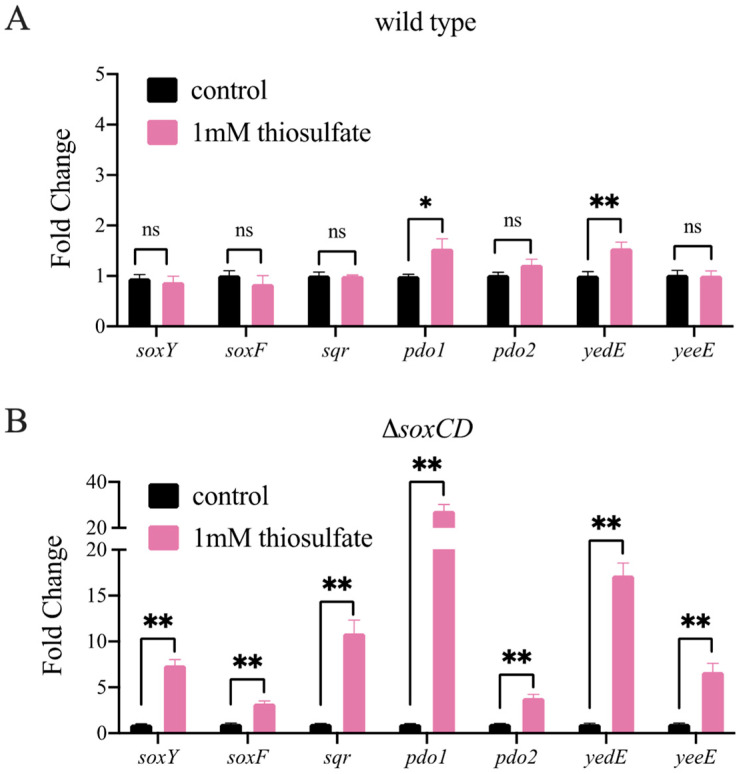
RT‒qPCR analysis of sulfur-related gene transcripts in thiosulfate (1 mM)-treated and untreated strains. Strains were grown in mineral medium until the *OD*_600_ reached 0.5. Then, 1 mM sodium thiosulfate was added to the medium. After 30 min of cultivation, RNA was extracted. A culture without the addition of sodium thiosulfate was used as the control. The relative expression levels of *soxY*, *soxF*, *sqr*, *pdo1*, *pdo2*, *yedE*, and *yeeE* in wild-type *C. pinatubonensis* JMP134 (**A**) and in Δ*soxCD* (**B**) were analyzed by RT‒qPCR. Each sample was repeated with three independent extractions of RNA. Results are presented as the mean ± SD. *: *p* < 0.05; **: *p* < 0.01. ns: not significant.

To confirm the function of these enzymes at the mRNA level, we detected the transcription of thiosulfate oxidation-related genes by real-time fluorescence quantitative polymerase chain reaction (PCR). The results showed that the addition of 1 mM thiosulfate did not significantly increase the expression of *soxY* in the wild-type strain. Moreover, the transcript levels of *pdo1* (1.3-fold), *pdo2* (1.1-fold), two H_2_S oxidases, *sqr* and *soxF*, and two putative sulfur-containing compound transporters, *yedE* (1.4-fold) and *yeeE* (0.9-fold), were not significantly increased (Figure 4A). These data indicated that the oxidation of thiosulfate by the complete Sox system does not require the participation of other enzymes. However, in Δ*soxCD*, the transcript levels of *soxY*, *pdo1,* and *pdo2* increased significantly by 7.5-, 31.3-, and 4.9-fold, respectively, with the addition of thiosulfate (Figure 4B), indicating that these two PDOs are involved in the oxidation of thiosulfate by the incomplete Sox system. In addition, the increase in the *soxY* expression level might indicate that more SoxY is needed for the carrying of sulfane sulfur. The transcript levels of *yeeE* and *yedE* increased by 7.8- and 18.4-fold in this knockout strain, respectively (Figure 4B), indicating that these two enzymes played an important role possibly in transporting sulfane sulfur from the periplasm to the cytoplasm. In addition, *sqr* and *soxF* expression levels were increased in this knockout strain (Figure 4B), indicating that H_2_S may have been produced temporarily.

In conclusion, these data further demonstrate that PDO and YeeE/YedE contribute to the oxidation of thiosulfate by the incomplete Sox system in *C. pinatubonensis* JMP134.

### 3.5. Distribution and Phylogenetic Analysis of SoxC, PDO and DsrAB

**Figure 5:**
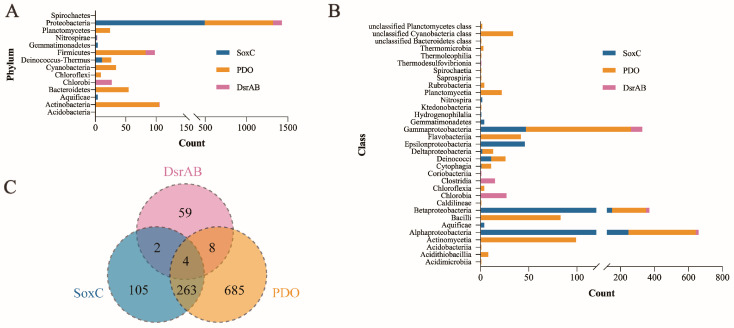
Distribution of homologous proteins of SoxC, PDO and DsrAB in selected bacteria. These homologous proteins were identified through BLASTp in a local database using the amino acid sequences of SoxC, PDO, and DsrAB as queries and further filtered by conserved domain analysis. The distribution of homologous proteins of SoxC, PDO, and DsrAB are shown at the phylum (**A**) and class levels (**B**). (**C**) The Venn diagram showing the number of species which harbored SoxC, PDO or DsrAB in selected bacteria.

We investigated the distribution of SoxC, PDO, and DsrAB in a microbial genomic protein sequence set of the genomes of 5013-type strain (updated on 26 June 2020). The result showed that the distribution of the three enzymes partially overlapped with each other. However, PDO was more widely distributed than SoxC, and both were more widely distributed than DsrAB. Among them, 516 SoxC homologous sequences were found in 374 species, mainly in the Proteobacteria and Deinococcus-thermous. An amount of 1154 PDO homologous sequences were found in 960 species, mainly in Proteobacteria, Plantomycetes, Firmicutes, Cyanobacteria, Deinococcus-thermous, Bacteroidetes, and Actinobacteria. There were only 150 homologous sequences of DsrAB, which were detected in 73 species, mainly in Proteobacteria, Firmicutes, and Chlorobi (Figure 5A,B). In addition, 263 species harbored both PDO and SoxC, but two strains harbored DsrAB, and eight strains harbored SoxC and PDO (Figure 5C).

Among the genomes of these 5013 species, 300 species belonging to Aquificae, Deinococcus-thermous, and Proteobacteria contained the complete Sox system. In contrast, only 62 species contained the incomplete Sox system and were mainly found in the Aquificae, Chlorobi, and Proteobacteria phyla, all of which lacked SoxC (Table 3, Tables S2 and S3). It is noteworthy that, among the 62 species with incomplete Sox systems, 21 species contained PDO, but not DsrAB, and were mainly found in the Betaproteobacteria, Alphaproteobacteria, Acidithiobacillia, and Gammaproteobacteria classes (Table 3 and Table S3).

**Figure 6:**
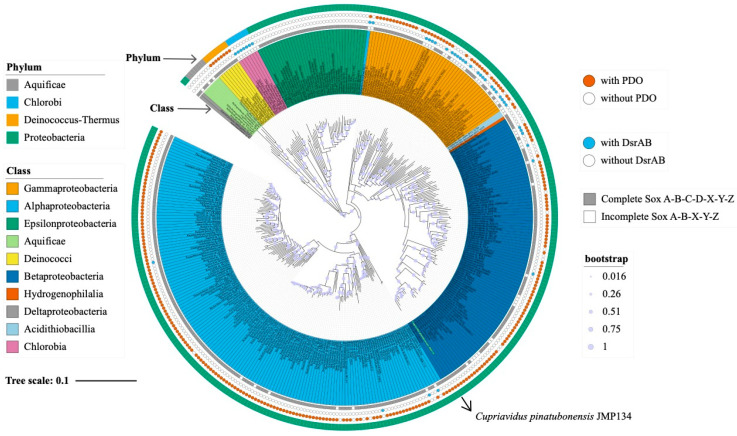
Phylogenetic tree based on 16S rRNA gene sequences. The neighbor-joining tree was calculated using an alignment of 362 representative 16S rRNA gene sequences of species containing complete and incomplete Sox systems. These sequences were aligned using the Muscle script, and the tree was built by using MEGA11 with pairwise deletion and p-distance distribution. Bootstrap analysis was performed with 1000 replicates. All 362 species are labeled, and different backgrounds represent lineage classes. Outside of the tree, the first ring (gray box) represents the integrity of the Sox system. The second (blue circle) and third ring (red circle) denote the presence or absence of PDO and DsrAB on the genome of a corresponding species, respectively. The outermost ring represents the lineage phylum of these 362 species. Circles on tree branches represent the percentage of bootstrap values.

We constructed a neighbor-joining phylogenetic tree of the 16S rRNA gene of 362 species with complete and incomplete Sox systems (Figure 6). The results showed that the integrity of the Sox system was not closely related to the taxonomic classification of populations, and a great degree of randomness was noted in the tree. In addition, in most clades of the tree, the species which harbored *pdo* or *dsrAB* seems to be scattered, suggesting that the pathway of thiosulfate oxidation by PDO or DsrAB with an incomplete Sox system may not have evolved synchronously with the taxonomic classification. However, few classes of organisms seem to contain enzyme systems with similar compositions. For example, all species in Chlorobia contain an incomplete Sox system and *dsrAB* but do not contain *pdo*. These microorganisms are also the model species for studying DsrAB at present. In addition, almost all bacteria in the class Epsilonproteobacteria contain the complete Sox system, but neither *pdo* nor *dsrAB* contain the same.

## 4. Discussion

Although emerging evidence suggests that sulfane sulfur has important physiological functions, sulfane sulfur must be maintained in a reasonable range, as excessive accumulation may cause cells to rupture [8]. The removal of excess sulfane sulfur is necessary for cell survival. In *C. pinatubonensis* JMP134, after thiosulfate was added, all strains, including the strains with knockout of sulfane sulfur oxidation-related genes (Δ*pdo12*, Δ*pdo12soxCD*), failed to permanently accumulate sulfane sulfur (Figure 2B). These findings suggest that the bacteria had adopted multiple pathways to avoid excessive accumulation of sulfane sulfur. SoxCD may be the preferred sulfane dehydrogenase of the Sox system. This enzyme uses sulfane sulfur bound to SoxYZ-S^−^ as the substrate to avoid the transmission of free sulfane [17]. Moreover, the enzyme oxidizes sulfane sulfur anaerobically and generates large amounts of electrons, which can serve as an important energy source for autotrophs [38]. PDOs are widely found in heterotrophic or chemoautotrophic bacteria and have important physiological functions [18,19]. In the heterotrophic bacterium *C. pinatubonensis* JMP134, PDO oxidize sulfane sulfur produced in the oxidation of H_2_S by SQR [3]. In this study, we revealed that PDO also oxidizes sulfane sulfur produced in the oxidation of thiosulfate by the incomplete Sox system (Figure 2). All these investigations expanded the range of substrate sources for PDO and indicated that PDO plays a key role in alleviating the pressure of excess sulfane sulfur. Finally, even after the knockout of all PDOs, the deletion strain failed to accumulate sulfane sulfur, and the excess sulfane sulfur was released by converting to volatilizable H_2_S gas (Figure 2D). Given that the actual substrate of PDO is GSSH, we speculate that H_2_S is produced through the spontaneous reaction GSSH+GSH→GSSG+H_2_S by the GSH/GSSG redox couple [39], which is consistent with the change in GSSG we observed (Figure 3B). GSH/GSSG is an important redox couple in bacteria that plays a critical role in protection against environmental stress [40]. Another possibility is that the protein-bound sulfhydryl group (R-SH), which may also produce H_2_S by a similar reaction mechanism, plays a role [41]. Further evidence is needed to determine the specific H_2_S production mechanism and direct sulfane sulfur acceptors. Thus, bacterium *C. pinatubonensis* JMP134 alleviated the pressure of sulfane sulfur accumulation through multiple pathways.

PDO generally works with the H_2_S dehydrogenase SQR to constitute the SQR/PDO system. The main function of SQR/PDO is to oxidize H_2_S to thiosulfate [26], and the generated thiosulfate can be further oxidized to sulfate by the Sox system [3]. Therefore, the Sox system often participates in the biological oxidation of H_2_S as a downstream enzymatic system of the SQR/PDO system. Conversely, either the complete Sox system transiently produced sulfane sulfur or the incomplete Sox system transiently produced H_2_S during the oxidation of thiosulfate (Figure 2), and these temporary sulfur compounds could be oxidized to thiosulfate by the SQR/PDO system and returned to the Sox system. Thus, the SQR/PDO system also participated in the oxidation process of thiosulfate to sulfate dominated by the Sox system. In addition, our bioinformatics analysis showed that SQR and PDO appeared in 66.3% and 76% of the species with a complete Sox system, respectively (Table S2). These findings further demonstrate that the Sox system is closely related to the SQR/PDO system. In summary, a diversified exchange in metabolites occurs between the SQR/PDO system and the Sox system.

## 5. Conclusions

In this paper, we revealed that *C. pinatubonensis* JMP134 alleviates the toxicity of sulfur accumulation through oxidation by PDO or H_2_S release, which provides more strategies for bacterial survival. This study expanded the understanding of the physiological function of PDO. One limitation of this study is that the transport mechanism of sulfane sulfur was not studied in detail, and more substantive direct evidence is needed to elucidate the properties and binding mechanism of sulfane sulfur receptors. In addition, the conclusions of this study are limited to the understanding of one strain of bacteria. The role of PDO in the biogeochemical sulfur cycle remains to be explored by metagenomes, metatranscriptomes, and metaproteomes of diverse environments and activity assays or protein-SIP experiments of more representative microorganisms.

## Figures and Tables

**Table 1:** Strains and plasmids used in this study.

Strains	Characteristics	Source
Wild-type strains		
*C. pinatubonensis* JMP134	Wild type	Our lab
*E. coli* S17-1	*recA pro thi hsdS*, RP4 *tra* functions, *supE44*	Invitrogen
Deletion strains		
Δ*soxCD*	*soxC* and *soxD* deleted in JMP134	This study
Δ*pdo12soxCD*	*pdo1, pdo2, soxC* and *soxD* deleted in JMP134	This study
Δ*pdo12*	*pdo1* and *pdo2* deleted in JMP134	[3]
Δ*pdo1soxCD*	*pdo1, soxC* and *soxD* deleted in JMP134	This study
Δ*pdo2soxCD*	*pdo2, soxC* and *soxD* deleted in JMP134	This study
Complementation strains		
Δ*pdo12soxCD::pdo1*	Δ*pdo12soxCD* with plasmid pBBR*-pdo1*	This study
Δ*soxCD:soxCD*	Δ*soxCD* with plasmid pBBR*-soxCD*	This study
Plasmids		
pBBR1MCS2	Kanamycin resistance, mob^+^, pBBR1 replicon, cloning vector	Qi qingsheng
pK18mobsacB	Widely used gene knockout vector, kanamycin resistance	Our lab
pBBR*-pdo1*	pBBR1MCS2 *containing pdo1*	This study
pBBR*-soxCD*	pBBR1MCS2 *containing soxCD*	This study

**Table 2:** Primers used in this study.

Target Gene	Primers	Sequence (5′-3′) ^d^
Deletion		
*soxC* and *soxD*	^a^ Up-f	CAGGAAACAGCTATGACATGATTACGAATTCACCGCCGGGTTTCTGTTG
	Up-r	CCTACCATCGGTTCCTGCAATGCCGTCTCCT
	^b^ Down-f	TTGCAGGAACCGATGGTAGGGTGGATTCTTGAG
	Down-r	TTCAGGATCCCCGGGTACCGAGCTCGAATTCTGCCATTGCTCTCTCCTGTTG
	^c^ V-f	GGTGTTCGGCTACACCATGT
	V-r	AGCTTTGCTCTCCGGCTAC
*pdo1*	Up-f	CAGGAAACAGCTATGACATGATTACGAATTAGACGATTACCTGGTCTACACCTTC
	Up-r	CAGCTGTTCGTACAGGCGCGTCAAATCCTTCTAT
	Down-f	CGCGCCTGTACGAACAGCTGATAGAAGGTTTGCAT
	Down-r	TTCAGGATCCCCGGGTACCGAGCTCGAATTGGCTGATGATGGAGAACGAAC
	V-f	TATTGGCTGCCATCTGCT
	V-r	GCTCTACAAGCTCAATGCG
*pdo2*	Up-f	CAGGAAACAGCTATGACATGATTACGAATTCGAGGTCGTAGCGGTAGTTG
	Up-r	ACACACATGAGCTATCTGAAGATTCCCCTCAAC
	Down-f	TTCAGATAGCTCATGTGTGTCTATCCGTGGTTAGC
	Down-r	TTCAGGATCCCCGGGTACCGAGCTCGAATTCCATTTCATCGAGGAATAGCGT
	V-f	ATGGCGTCCCAATCCAGCTT
	V-r	TTGCCTGGAGAGTGGCTTTG
Complementation		
*soxC* and *soxD*	Forward	CACACAGGAAACAGCTATGCAGGAACGCACACC ^e^
	Reverse	TTCCATTCGCCATTCACTATTTTGCCTCAAGAATCCA ^e^
*pdo1*	Forward	CACACAGGAAACAGCTATGACACCGACCATGCCAAG ^e^
	Reverse	TTCCATTCGCCATTCATCAGAGGGCGTTGAGGGG ^e^
Linearization		
pBBR1MCS2	Forward	TGAATGGCGAATGGAAATTGTAAG
	Reverse	AGCTGTTTCCTGTGTGAAATTGTTATC
RT‒PCR		
*soxY*	Forward	GAGTGGAACAAGACCGCTTT
	Reverse	ATCGCGATCTGCTCGGTATC
*pdo1*	Forward	CACGCTCTACCGTTCCATCA
	Reverse	CACGTGGATGTTGTTCTCGC
*pdo2*	Forward	CATGCCCATGCCGACCACATC
	Reverse	CGTGCCGAAGGTGAGCGTATC
*sqr*	Forward	GCGTGGTGAAGTACGAACAA
	Reverse	AGGTCGTAGCGGTAGTTGGA
*soxF*	Forward	GCGTGAGTGGAGCGGACATG
	Reverse	GATTGGACAGCGGACACGAGAC
*yedE*	Forward	TTGCACGAAGACGGTCAGGAAAC
	Reverse	CACGGCGTGTGCGGAATCTC
*yeeE*	Forward	GGAAAGCCACCAGCCCGATG
	Reverse	CCGCCAAGGTGCAGGGATTC

^a^: The primers used to clone the upstream sequence of the target gene. ^b^: The primers used to clone the downstream sequence of the target gene. ^c^: The primers used to verify the mutants. ^d^: Underlined text represents the overlapping sequences with plasmid pK18mobsacB. ^e^: Underlined text represents the overlap sequences with plasmid pBBR1MCS2.

**Table 3:** The number of species possessing the complete or incomplete Sox system.

Phylum	Class	Complete Sox System			Incomplete Sox System		
		Total	PDO	DsrAB	Total	PDO	DsrAB
Aquificae	Aquificae	4	0	0	3	0	0
Chlorobi	Chlorobia	0	0	0	7	0	7
Deinococcus-Thermus	Deinococci	8	8	0	0	0	0
Proteobacteria	-	288	230	8	52	36	27
	Acidithiobacillia	0	0	0	2	2	0
	Alphaproteobacteria	146	140	2	8	5	3
	Betaproteobacteria	75	63	1	20	16	5
	Deltaproteobacteria	2	0	0	0	0	0
	Epsilonproteobacteria	35	0	0	1	0	0
	Gammaproteobacteria	29	27	5	21	13	19
	Hydrogenophilalia	1	0	0	0	0	0
Total		300	238	8	62	36	34

## Data Availability

The names of the repository/repositories and accession number(s) can be found in the article/supplementary material. The mutants of *C. pinatubonensis* JMP134 constructed in this study were stocked in our lab and will be made available upon request.

## Supplementary Materials

The following supporting information can be downloaded at: https://www.mdpi.com/article/10.3390/metabo13020218/s1, Figure S1: Thiosulfate oxidation by complemental strains of *C. pinatubonensis* JMP134 mutants; Figure S2: Volatilization of H_2_S upon oxidation of thiosulfate by three strains detected by lead acetate test strips; Table S1: Selected genomes of type strains; Table S2: Species harboring the complete Sox system; Table S3: Species harboring the incomplete Sox system.
