# Supplementary material for: Cupriavidus pinatubonensis JMP134 Alleviates Sulfane Sulfur Toxicity after the Loss of Sulfane Dehydrogenase through Oxidation by Persulfide Dioxygenase and Hydrogen Sulfide Release
Source: Metabolites. 2023 Feb 2;13(2):218. doi: 10.3390/metabo13020218 (PMC9959259; doi:10.3390/metabo13020218)
Supplement: Supplementary file 1 [file metabolites-13-00218-s001.zip › Supplemental material 10.pdf]

Supplemental material

Cupriavidus pinatubonensis JMP134 alleviates sulfane sulfur toxicity after the loss of sulfane dehydrogenase through oxidation by persulfide dioxygenase and hydrogen sulfide release

Yufeng Xin[1,†,*], Yaxin Wang[1,†], Honglin Zhang[1], Yu Wu[1], Yongzhen Xia[2], Huanjie Li[3], Xiaohua Qu[1,*]

[1] College of Life Sciences, Qufu Normal University, Qufu, 273165, P. R. China.

[2] State Key Laboratory of Microbial Technology, Shandong University, Qingdao, 266237, P. R. China.

[3] School of Medicine, Cheeloo College of Medicine, Shandong University, Jinan, 250012, Shandong, China.

[*] Corresponding author. Xiaohua Qu, Email: anty214@163.com; Yufeng Xin, E-mail: xinyufeng@qfnu.edu.cn; Tel: +86 15562345068.

[†] These authors have contributed equally to this work

This supplemental document contains:

Figure S1: Thiosulfate oxidation by complemental strains of *C. pinatubonensis* JMP134 mutants.

Figure S2. Volatilization of H_2S upon oxidation of thiosulfate by three strains detected by lead acetate test strips

Table S1 Selected genomes of type strains

Table S2 Species harboring the complete Sox system

Table S3 Species harboring the incomplete Sox system

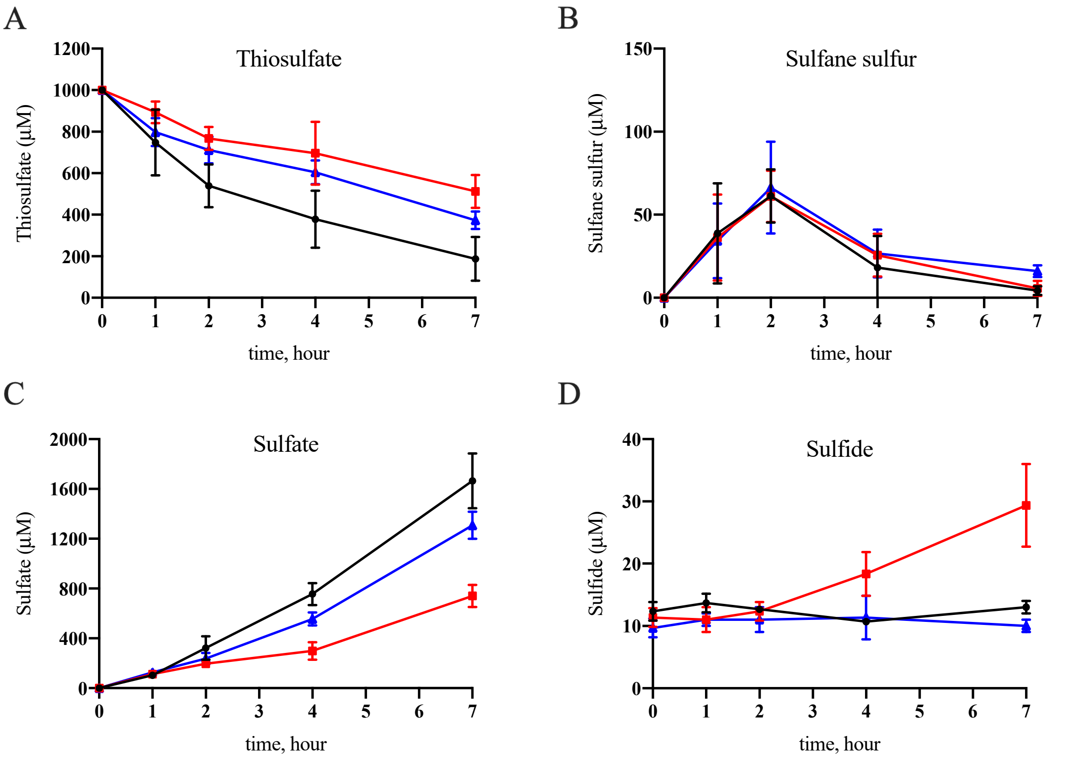

Figure S1. Thiosulfate oxidation by complemented strains of *C. pinatubonensis* JMP134 mutants. Cells were harvested, wash and re-suspended at OD_{600} of 2.0 in 100 mM HEPES buffer, pH 7.0. 1 mM thiosulfate and 0.5 mM zinc acetate were added together to initiate the reaction. Thiosulfate, sulfane sulfur, sulfate and sulfide were determined at different time points. *C. pinatubonensis* JMP134 (●), Δ*pdo12soxCD::pdo1* (■) and Δ*soxCD::soxCD* (▲). All data are average of at least three samples with standard deviation (error bar).

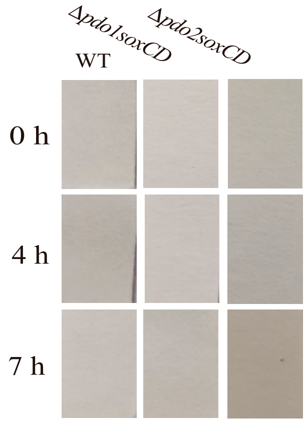

Figure S2. Volatilization of H_2S upon oxidation of thiosulfate by three strains detected by lead acetate test strips
